# Predictors of time to relapse in amphetamine-type substance users in the matrix treatment program in Iran: a Cox proportional hazard model application

**DOI:** 10.1186/s12888-016-0973-8

**Published:** 2016-07-26

**Authors:** Maryam Moeeni, Emran M. Razaghi, Koen Ponnet, Fatemeh Torabi, Seyed Ali Shafiee, Tahereh Pashaei

**Affiliations:** 1 Health Management and Economics Research Center, Isfahan University of Medical Sciences, Isfahan, Iran; 2 Iranian National Center for Addiction Studies (INCAS), Tehran University of Medical Science (TUMS), Tehran, Iran; 3 Department of Psychiatry, School of Medicine, Tehran University of Medical Sciences, Tehran, Iran; 4 Department of Communication Studies, Media, ICT/Interpersonal Relations in Organizations and Society (MIOS), University of Antwerp, Sint-Jacobsstraat 2, 2000 Antwerp, Belgium; 5 Department of Communication Studies, Research Group for Media & ICT (MICT), Ghent University, Korte Meer 11, 9000 Ghent, Belgium; 6 Higher Institute for Family Sciences, Odisee, Huart Hamoirlaan 136, 1030 Brussels, Belgium; 7 Antwerp Maritime Academy, Noordkasteel Oost 6, Antwerp, 2030 Belgium; 8 Department of Demography, Faculty of Social Sciences, University of Tehran, Tehran, Iran; 9 Department of Neurosciences and Addiction Studies,School of Advanced Technologies in Medicine, Tehran University of Medical Sciences, Tehran, Iran; 10 Social Determinants of Health Research Center, Kurdistan University of Medical Sciences, Sanandaj, Iran; 11 Department of public health, School of Health, Kurdistan University of Medical Sciences, Sanandaj, Iran

**Keywords:** ATS dependence, Matrix model, Treatment program, Relapse, Survival analysis

## Abstract

**Background:**

The aim of this study was to determine which predictors influence the risk of relapse among a cohort of amphetamine-type substance (ATS) users in Iran.

**Methods:**

A Cox proportional hazards model was conducted to determine factors associated with the relapse time in the Matrix treatment program provided by the Iranian National Center of Addiction Studies (INCAS) between March 2010 and October 2011.

**Results:**

Participating in more treatment sessions was associated with a lower probability of relapse. On the other hand, patients with less family support, longer dependence on ATS, and those with an experience of casual sex and a history of criminal offenses were more likely to relapse.

**Conclusion:**

This study broadens our understanding of factors influencing the risk of relapse in ATS use among an Iranian sample. The findings can guide practitioners during the treatment program.

## Background

Amphetamine-type substance (ATS) refers to the varying forms of stimulant drugs such as methamphetamine, amphetamine, methylenedioxymethamphetamine (MDMA, also known as “ecstasy”), methcathinone, and ephedrine. ATS is the second most commonly used illicit drug type worldwide after cannabis [1, 2]. The non-medical use of ATS poses potential health problems. Several studies reported that ATS dependence contributes to physical and psychiatric diseases, including heart damage, brain damage, impaired thinking, mood disturbance, aggression, violence, cognitive impairment, psychotic symptoms, poisoning, and even death [3]. Furthermore, ATS dependence is associated with increased financial problems, delinquency, and communication difficulties [3, 4]. In Iran, drug dependence has become more and more of both a social and a health problem. While opioid consumption has a long history in Iran, the use of stimulant drugs such as ATS started to rise around 2000 [5, 6]. The growing rate of stimulant use in Iran is rooted in the easy availability of ATS, increased prices of opium, curiosity for this new type of drug, public ignorance about the dangers of ATS dependence, and expectation of sexual benefits during ATS use [5].

However, little is known about the rate of stimulant drug use in Iran, mainly because it is a relatively new phenomenon in this country [7]. Some recent studies have suggested a large number of stimulant users [7, 8]. For instance, in a study conducted by Hajiabdolbaghi and colleagues in which the sexual behaviour among a group of at-risk women in Tehran was investigated, the authors found that 18.5 % of people aged between 15 and 25 years old reported they had used illicit substances, including ATS [9]. In addition, cases of cardiomyopathy, paranoia, suicide, and homicide resulting from ATS use have become more prominent in medical and psychiatric emergencies in Iran [5]. Furthermore, because these stimulant drugs can be injected, they have become associated with the transmission of infectious diseases such as HIV/AIDS and hepatitis, both of which are already major health problems among the drug dependents in Iran [10, 11].

As a response to the emerging health and social crises related to ATS, in 2007 the Iranian National Center for Addiction Studies (INCAS) started a pilot of the Matrix program as a cognitive-behavioral intervention and has continued to focus on this problem since that time. The program was named after the Matrix Center in Southern California, the treatment center in which the program was initially founded [12]. Originally developed for the treatment of cocaine, the Matrix model is a “multi-element package of therapeutic strategies” that includes cognitive-behavioral therapy, research on relapse prevention, motivational interviewing strategies, psycho-educational information, and 12-step program involvement [13]. Several studies have supported that cognitive-behavioral interventions are effective treatments for stimulant drug dependencies [12, 13].

Relapse is however a major difficulty in addiction treatment. Indeed, others studies confirmed the high rate of relapse in ATS users [14–18]. For example, one study conducted by Brecht and Herbeck (2014) showed that 61 % of the methamphetamine users relapsed within 1 year following treatment discharge [18]. Relapse is not the result of a single factor and is usually caused by a combination of demographic and physiological characteristics, situational and socio-cultural features, and treatment characteristics [18–22]. While, most previous studies investigated correlates of relapse into drug use among opioid dependents [19, 20, 23–29], few studies focused on factors related to relapse among ATS users including demographic predictors such as age [22, 28]; gender [30]; education attainment [22]; receiving social support [28]; patients’ psychological comorbidities [22, 28, 31]; history of prison [28]; and drug-use characteristics, such as age of starting ATS use [20], history of drug injection [32], selling substances [20, 33], duration of lifetime ATS dependence [31], and treatment characteristics (including age of participating in treatment program [22] and type of treatment [12, 20, 34]). However, at this point there is no sufficient evidence for pattern of time to relapse among ATS users. Thus, the goal of this study is to examine covariates associated with time to relapse in ATS users who participated in the Matrix treatment program focussing specially on examination of the contribution of the Matrix program for reducing the risk of relapse. We ran a multivariate Cox proportional hazards regression model as a semi-parametric survival model to study the factors associated with time to relapse into ATS usage. The results can help to predict which clients are more at risk for relapse. As such, the findings may have wider relevance to therapists and practitioners who have to identify people who are susceptible to relapse and, as such, prevent them from actual relapse by offering them additional services.

## Method

### Participants

The study used a retrospective cohort design. The data were collected from March 2010 until October 2011. A total of 168 ATS users entered the Matrix treatment program run by INCAS. However, 39 clients who discontinued their contact with the therapists at INCAS were excluded from the analyses because there was no follow-up data for them. To test for possible biases, the 39 clients with missing data were compared with the rest of the sample. As shown in Tables 1 and 2, no significant differences were found between the 39 clients and the rest of the sample regarding any of the background variables. This indicated that the 39 clients with missing follow-up data were not systematically different from those who completed the program. Of the 129 participants who completed the follow-up period, there was only one woman. This female participant was excluded from the sample for statistical reasons. Thus, our analyses of time to relapse were conducted on 128 males. This study was approved by the ethical committee of Tehran University of Medical Sciences. The program was explained to each participant, and informed consent was obtained from all patients prior to their participation in the study.

**Table 1:** Demographic and psychological characteristics of the sample

	Patients included (*n* = 128)		Patients excluded (*n* = 39)		
	*n*	Mean/%	*n*	Mean/%	*p* value
Demographic variables					
Age at the time of admission	128	30.10	39	30.00	.99^b^
High school graduate					
Yes	78	60.93 %	24	61.53 %	.99^a^
No	50	39.06 %	15	38.46 %	
Marital status					.89^a^
Single	56	43.75 %	18	46.15 %	
Married	45	35.16 %	12	30.77 %	
Divorced	27	21.09 %	9	23.08 %	
Employment status					.95^a^
Full time	56	43.75 %	16	41.03 %	
Part time	24	18.75 %	8	20.51 %	
Unemployed	48	37.50 %	15	38.46 %	
Psychological variables					
Aggression					.99^a^
Yes	73	57.03 %	22	56.41 %	
No	55	42.97 %	17	43.59 %	
Criminal offences					.79^a^
Yes	36	28.15 %	12	30.77 %	
No	92	71.88 %	28	71.80 %	
Casual sex					.99^a^
Yes	100	78.13 %	29	74.36 %	
No	28	21.88 %	10	25.64 %	

Note

^a^chi-square test; ^b^t-test

**Table 2:** Drug related characteristics of the sample

	Patients included (*n* = 128)		Patients excluded (*n* = 39)		
	*n*	Mean/%	*n*	Mean/%	*p* value
Age at first time of drug use	128	22.00	39	21.32	.68^b^
Duration of Addiction (in years)	128	5.20	39	5.25	.99^b^
Duration of amphetamines dependence (in years)	128	2.20	39	2.02	.79^b^
Family support					.99^a^
Yes	32	25.00 %	9	23.08 %	
No	96	75.00 %	30	76.92 %	
Having (at least) an addict in the family					.79^a^
Yes	46	35.94 %	13	33.33 %	
No	82	64.06 %	26	66.67 %	
Injection experience					.59^a^
Yes	26	20.31 %	9	23.08 %	
No	102	79.69 %	30	76.92 %	
Poly substance abuse					.63^a^
Yes	110	85.94 %	32	82.05 %	
No	18	14.06 %	7	17.95 %	
Amphetamines dependence as the primary diagnosis					.99^a^
Yes	113	88.29 %	34	87.18 %	
No	18	11.72 %	5	12.82 %	
Treatment experience					.99^a^
Yes	95	74.22 %	29	74.36 %	
No	33	25.78 %	10	25.64 %	
A history of residential treatment in camps					.76^a^
Yes	78	60.94 %	22	56.41 %	
No	50	39.06 %	17	43.59 %	

Note.

^a^chi-square test; ^b^t-test

### Study design

According to INCAS policy, a fee of 1,000,000 rials (approximately 35 USD, with an official minimum wage of 300 USD) was mandatory prior to admission to the program. The inclusion criteria of the treatment program were as follows: (1) being dependent to ATS, according to the Diagnostic and Statistical Manual of Mental Disorders, Fourth Edition (DSM-IV) and (2) being at least 18 years old at the time of admission. The authors collected information on the patients over 8 months. Patients were monitored during a 2-month treatment program and after the treatment program, for a follow-up period of 6 months. All patients gave the information that was required; hence, there were no missing data on the 128 patients included in the analyses.

Relapse criteria were defined as both a positive urinary ATS test and a return to ATS use reported by the patients or their family members. Patients had regular urine analysis tests, i.e., twice in the 1st month after starting the treatment program and once in the following months. At the end of the follow-up period, the researchers contacted the family members of the patients and asked them about the patients’ relapse during the treatment program and the follow-up period. Of the 128 patients, 49 patients (38 %) had not relapsed by the end of the follow-up period.

### The Matrix program

The treatment program consisted of up to 29 sessions of individual counselling, but the average number of sessions completed was 13. All sessions were held at INCAS in the 2 months after starting the treatment program for each patient. Since INCAS is an outpatient centre, participating in each counselling session was voluntary. Consultants were experienced psychologists working as INCAS staff. All consultants had been trained for about 80 hours to obtain enough knowledge of and experience with the Matrix model before starting the program. During each session, a consultant talked with the client about a single topic. The aim was to help patients cope with difficulties they faced during recovery. The topics included deal with cravings, impulsive sexual behaviors, lack of energy, dealing with families’ distrust, sense of shame or guilt, unwanted behaviors, and emotions resulting in drug use. Further, the consultants identified opportunities that facilitated patients’ treatment including motivation, honesty, and employment. Patients were also required to do some homework in order to improve their social skills.

### Data collection and study variables

In this study, the outcome variable was time to relapse into ATS use since starting the treatment program. Several demographic and psychological characteristics of the respondents were collected at the intake. Data were collected on sex, age at the time of admission to the program, high school graduation (yes/no), marital status (single, married, divorced), and employment status (full time, part time, unemployed). With regard to the psychological characteristics, data were collected on aggression experiences (yes/no), history of criminal offences (yes/no) and history of casual sexual relationships (yes/no) during the 6 months before admission to the treatment program.

Furthermore, different predictors that might be associated with the risk of relapse into ATS use were analysezed. The main predictor of interest was the Matrix treatment program, assessed by the number of consultation and training sessions that each patient attended. In addition, data were collected on age at first drug use, drug injection experience (yes/no), duration of drug dependence in general (in years), duration of ATS dependence (in years), having another addicted family member (yes/no), self-reported family support (yes/no), and poly-substance use (yes/no), which was defined as using opioids —especially opium and heroin— crack, and cannabis, as well as alcohol, during the period of ATS dependence (not lifetime) at least 4 times a week.

### Data analysis

The inclusion of the study variables allowed the researchers to predict which clients were more at risk for relapse. To do so, we applied a Cox proportional hazards regression model as a semi-parametric method of survival analysis. Survival models are preferred over classic regression models (such as a logistic regression) because the former analyzes both the time elapsed before an event and the probability of occurrence of the event. The Cox model is based on the assumption of the proportionality of hazards, meaning that the hazard ratio of each variable does not change over time [35].We assessed this assumption based on a goodness of fit test. Data analyses were performed using Stata version 11.

## Results

### Description of the sample

Descriptive characteristics of the 128 patients are shown in Tables 1 and 2. At the time of admission, the mean age of the study participants was 30.1 years. Thirty-nine present of the patients had dropped out of high school. With regard to marital status, about 44 were single, 35 were married, and 21 were divorced. Of the 63 % of the sample who had a job, about 44 % worked full time. The mean age of starting drug use was 22 years. The median lifetime periods of drug dependence in general and ATS dependence in particular before intake were 48 and 24 months, respectively.

Few patients (20 %) had practised drug injection, but most (86 %) had engaged in poly-substance use before starting the treatment program. For many of the participants (88 %), ATS dependence was the primary diagnosis. According to the patients’ self-reports, 57 had a history of aggressive behaviors, and 28 % had been arrested at least once for criminal offences. However, no one had been involved in any violent criminal activities. Almost 78 % reported having had casual sexual relationships. A minority of participants (25) reported that they were receiving passable or strong family support, and around 36 % said that at least one of their family members was also drug dependent. The majority of patients (74 %) had previously enrolled in other stimulant drug treatment programs, and about 61 % had tried residential treatment programs. Most of the patients (98 %) were dependent on methamphetamines, while 2 % were dependent on ecstasy. Nearly 62 % of the patients relapsed into ATS dependence during the treatment program or in the 6-month follow-up period.

**Fig. 1:**
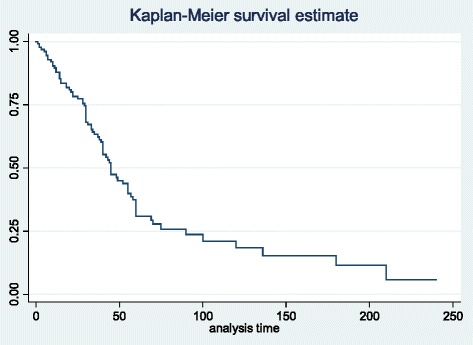
Kaplan-Meier estimate of time to relapse

Figure 1 illustrates Kaplan–Meier estimates of relapse rates among the patients. The rates of time to relapse for 30 days, 90 days, and 180 days from the time of admission were 68, 24, and 11 %, respectively.

### Multivariate analysis

A normal Cox regression was performed to examine the relationship between potential predictors related to risk of relapse.

**Fig. 2:**
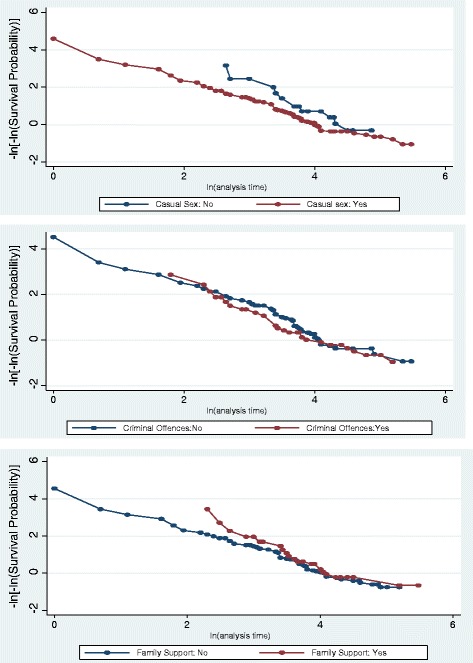
log-log survival estimates against the log of time

As indicated in Table 3 and Fig. 2, all significant predictors in the Cox model satisfied the proportional hazard (PH) assumption according to the goodness-of-fit test (based on the Schoenfeld residuals), pointing us toward the Cox proportional hazards model. As shown in Table 4, five predictors —including Matrix treatment attendance, casual sex habits, criminal offences, family support, and lifetime duration of ATS dependence— were found to be significant. More specifically, estimated hazard ratios (HRs) suggest that, on average, the rate of relapse into the drug reduced by 18 per each additional session of consultation and training attended (HR = .82, *p* = 0.000). In addition, on average, patients with a history of casual sex habits had a shorter time to relapse compared to the other group (HR = 2.03, *p* = 0.025). The rate of relapse to drug among patients with a history of criminal offence was .59 higher compared to the other group (HR = 1.59, *p* = 0.033). Furthermore, passable or strong family support was a predictor of longer time to relapse (HR = 0.52, *p* = 0.003). Finally, the risk of relapse into drugs increased .02 per every one year of ATS dependence before intake to the Matrix program (HR = 1.02, *p* = 0.001).

**Table 3:** Test of proportional hazard assumption

Variable	rho	chi2	df	prob > chi2
The Matrix treatment program (in sessions)	0.05	0.61	1	0. 44
Casual sex	−0.07	0.33	1	0.57
Criminal offences	0.15	1.46	1	0.23
Duration of amphetamines dependence (in years)	0.01	0.00	1	0.96
Family support	0.01	0.00	1	0.95
Global test		1.96	5	0.86

**Table 4:** Cox PH model of stimulants relapse predictors

Variable	Hazard ratio	Robust SE	Confidence of interval (95 %)	*p*-value
The Matrix treatment program (in sessions)	.82	.04	.75 .9	0.000
Casual sex				
Yes	2.03	.65	1.09 3.79	0.025
No	-	-	- -	-
Criminal offences				
Yes	1.59	.35	1.04 2.45	0.033
No	-	-	- -	-
Duration of Amphetamines dependence (in years)	1.02	.01	1.01 1.03	0.001
Family support				
Yes	.52	.12	.33 .80	0.003
No	-	-	- -	-

## Discussion

To the best of our knowledge, this is the first study conducted among ATS users in Iran in which the predictors of time to relapse into ATS use were examined while undergoing the Matrix treatment program, carried out at INCAS. This information can fill in important gaps in our knowledge about a drug that has only recently become a major area of concern in Iran.

In this study, over half of the participants relapsed (61 %). This finding is consistent with similar studies that confirmed a high rate of relapse into ATS use after treatment discharge [14–18]. Using a Cox proportional hazards model, a greater number of Matrix sessions was found to be associated with a lower risk of relapse into ATS use. The more consultation and training sessions that patients attended, the longer time to relapse they had. The results thus support the value of treatment sessions in reducing relapse risk and, in particular, of encouraging clients to attend as many of the treatment sessions as they can. Although the findings of one previous study suggested the Matrix treatment program was related to longer retention time [12], it did not focus on the exact effect of attendance in consultation sessions.

An influential predictor of shorter time to relapse into ATS use was casual sex history. Patients engaging in casual sex prior to the treatment enrolment faced higher risk of relapse. This finding is in line with other studies on different kinds of drug dependencies [36, 37]. This effect may be related to this idea that being in a less stable relationship results in less compliance with treatment programs. In addition, the motivation for re-experiencing sex under influence of stimulants might relate to this result. Furthermore, our results are consistent with previous studies in which it was found that a history of criminal offences before intake increases the risk of treatment dropout [38, 39].

Interestingly, this study found that strong family support was associated with longer time to relapse (i.e. they were less at risk of relapse to ATS use). This finding is in line with the results of other studies focussing on different treatment for various types of drug dependencies [40, 41]. Patients receiving family support have usually enhanced their mental health, which determines better treatment outcomes [42]. Further, family support for regular attendance of treatment sessions, the family’s role in coping with drug cravings and/or providing a supporting environment for treatment at home, and patients’ desire to satisfy their families are other explanations for good treatment outcomes.

Furthermore, another factor that was associated with a shorter time to relapse was duration of lifetime period of ATS dependence before starting the treatment. This finding is consistent with the results of other studies focussing specially on cocaine addiction [43–45] and suggests that people who have been drug users for a long period of time have more difficulty remaining abstinent than others [31, 44].

None of the demographic variables were significant associated with relapse to ATS in this study While, some previous studies have shown associations between demographic variables and relapse to ATS [22, 28], two other studies found results similar to our study. One explanation may be the spread of drug abuse among people with different age, occupation, education, and other backgrounds.

It is interesting that nearly all of the patients who joined our treatment program were males. There are several explanations for this gender discrepancy. First, ATS dependence is not common among Iranian women [46, 47]. Second, less social and family support is given to drug-dependent women in Iran. In general, women might feel greater stigma than men about treatment, providing the women with fewer opportunities to participate in treatment programs [48]. Finally, drug-dependent women might experience more difficulties paying for the treatment sessions than men because of the high unemployment rate among Iranian women.

This study was subject to some limitations. First, almost all the information was collected according to the patients’ self-reports. As such, comorbidities specified along with their drug dependence diagnosis were not based on a systematic professional diagnosis. Furthermore, patients are not always aware of their drug-use characteristics such as time of initiation of dependence. Therefore, a better but more time-consuming approach to access these variables would be to ask about each criterion of dependence separately and the time of initiation of each criterion. Second, all study members were male; therefore, the results cannot be generalised to both sexes. Third, most information for both included and excluded clients was collected at the baseline, and we did not consider the time varying covariates. Finally, several variables related to treatment program, such as the quality of services and rapport between patients and therapists, that might influence the relapse rate were not considered in this study because of a lack of any information on which to test them.

## Conclusion

Participating in more treatment sessions is associated with less risk of relapse. Still, more attention, help, and consultations should be given to patients with less family support, longer dependence on ATS, and those with a history of casual sex and/or of criminal offences. The findings can guide practitioners during the treatment program.

## Abbreviations

AIDS, acquired immune deficiency syndrome; ATS, amphetamine-type substance; DSM-IV, Diagnostic and Statistical Manual of Mental Disorders, Fourth Edition; HIV, human immunodeficiency virus; HR, hazard ratios; INCAS, Iranian National Center of Addiction Studies; MDMA, methylenedioxymethamphetamine; USD, United State Dollar
